# Symptoms of post traumatic stress disorder and their relationship with the fear of COVID−19 and COVID−19 burden among health care workers after the full liberalization of COVID−19 prevention and control policy in China: a cross-sectional study

**DOI:** 10.1186/s12888-023-05399-z

**Published:** 2023-12-05

**Authors:** Huan Liu, Ningying Zhou, Zhiqing Zhou, Xiubin Tao, Yan Kong, Ming Zhang

**Affiliations:** 1 Department of Hemodialysis, The First Affiliated Hospital of Wannan Medical College (Yijishan Hospital of Wannan Medical College), Wuhu, China; 2 Wuxi School of Medicine, Wuxi Maternity and Child Health Care Hospital, Jiangnan University, Wuxi, China; 3 Department of Nursing, The First Affiliated Hospital of Wannan Medical College (Yijishan Hospital of Wannan Medical College), Wuhu, China; 4 School of Humanities and Social Science, University of Science and Technology of China, Hefei, China; 5 School of Innovation and Entrepreneurship, Wannan Medical College, Wuhu, China; 6 School of Educational Science, Anhui Normal University, No. 1 Beijing East Road, Wuhu, 241000 China

**Keywords:** PTSD symptoms, COVID-19, Liberalization, Policy, China

## Abstract

**Background:**

Over the past three years, the COVID-19 pandemic has brought an overwhelming impact on China**’**s hospital system and health care workers, which can lead to post traumatic stress disorder (PTSD) symptoms. Previous research has shown that the COVID-19 pandemic had long-term adverse effects on the mental health of health care workers. Indeed, PTSD symptoms have emerged as one of the significant mental health issues for health care workers arising from the COVID-19 pandemic. Therefore, we conducted this cross-sectional survey to investigate the prevalence of PTSD symptoms in health care workers and its relationship with the fear of COVID-19 and the COVID-19 burden after the full liberalization of COVID-19 prevention and control policy in China.

**Methods:**

This study was conducted three years after the global COVID-19 pandemic (January 15 to January 16, 2023). This study was conducted via the Wenjuanxing platform and used the Chinese versions of the scales PC-PTSD-5, COVID-19 Anxiety Scale (FCV-19S), Social Support Scale, COVID-19 Stress Scale, GAD-2, and PHQ-2.

**Results:**

The prevalence of PTSD symptoms in health care workers was 24.3% (232/955). depression(*P* < 0.001), anxiety(*P* < 0.05), the fear of COVID-19(*P* < 0.001), and COVID-19 burden(*P* < 0.001) were highly correlated with PTSD symptoms in health care workers. Social support(*P* < 0.05) was a protective factor of PTSD symptoms.

**Conclusions:**

This survey shows that PTSD symptoms were highly prevalent among Chinese health care workers after the COVID-19 pandemic. Governments and leaders of medical institutions should, through psychological interventions, address the current situation of PTSD symptoms among health care workers and develop targeted programs and strategies to reduce their psychological problems.

## Introduction

On January 30, 2020, the World Health Organization (WHO) declared the COVID-19 outbreak as a public health emergency of international concern (PHEIC) [1]. On March 11, 2020, the World Health Organization (WHO) declared the COVID-19 outbreak a global pandemic. In the early days of the COVID-19 pandemic, United Nations Secretary-General Antonio Guterres issued a broad call for governments worldwide to prioritize the mental health of frontline health workers and provide them with mental health services [2]. Over the past three years, the COVID-19 pandemic has overwhelmingly impacted China**’**s hospital system and health care workers.

A recent review by Lee et al. [3] identified the studies that have investigated post traumatic stress disorder (PTSD) symptoms specifically among healthcare workers (HCWs). However, despite the extensive research on PTSD symptoms in this field, there are inconsistent findings, particularly among those working in frontline settings, as highlighted by Lee et al. [3], which inconsistencies warrant further investigations.

As previously reported in the research literature, complications of COVID-19 include mental health issues such as PTSD symptoms [4]. PTSD is a mental health problem associated with stressful events after experiencing or witnessing a life-threatening traumatic event that has significantly burdened individuals and society for an extended period [5]. PTSD is a psychological response to a traumatic event, which mainly includes re-experience, avoidance, negative changes in mood and hypervigilance [6, 7]. It is a mood disorder dominated by anxiety and fear, which can lead to depression, helplessness, memory impairment, and reduced work quality [8]. Previous studies [9, 10] have shown that direct and indirect exposure to trauma can lead to PTSD symptoms.

Indeed, PTSD symptoms have emerged as one of the significant mental health issues for health care workers arising from the COVID-19 pandemic. Studies have shown that when individuals experience major public health emergencies, they are prone to anxiety, depression, PTSD symptoms, and many other psychological problems [11, 12]. The COVID-19 pandemic has brought an overwhelming impact on health care workers. A growing body of research literature shows high PTSD symptom levels among health care workers during the COVID-19 pandemic [13–18]. Working in high-risk positions, being in contact with infected people, and being isolated are known risk factors for PTSD symptoms [19–21].On November 11 and December 7, 2022, the comprehensive group of the Joint Prevention and Control Working Mechanism of The State Council, in response to the novel coronavirus infection, successively issued the Notice on Further Optimizing the Prevention and Control Measures of the Novel Coronavirus ( “Twenty Measures”) and the Notice on Further Optimizing and Implementing the Prevention and Control Measures of the Novel Coronavirus ( “New Ten Measures”) [22, 23]. In order to ensure the normal order of production and life in society, the “Notice” clearly requires that all localities are strictly prohibited from arbitrarily closing schools, suspending classes, suspending work and production, etc., and localities are not allowed to increase control. After the full liberalization of COVID-19 prevention and control policies, high levels of exposure to COVID-19 patients mean the potential for a mass traumatic event with unprecedented impacts on healthcare workers**’** mental health. Studies have found that health workers working on the front line of the pandemic had reported higher levels of distress [20, 24]. Previous studies have shown that PTSD symptoms are a psychiatric disorder that occurs after a frightening or life-threatening traumatic event, leading to long-term adverse changes in cognition and mood [25–27]. Studies in Singapore found that during the COVID-19 pandemic, the incidence of PTSD symptoms in nurses was 18% [28]. Studies have found that PTSD symptom levels among health care workers remain high six months after the pandemic [13] and one year after the pandemic [29]. Therefore, further research on the prevalence of PTSD symptoms among health care workers two years after the pandemic is needed.

Previous findings have pointed to an increased risk of PTSD symptoms among health care workers during the COVID-19 pandemic. In the context of the full liberalization of COVID-19 prevention and control policies among people affected by the COVID-19 pandemic, particular attention should be paid to health care workers infected with COVID-19. We suspect that health care workers infected with COVID-19 will likely develop PTSD symptoms later in the COVID-19 pandemic. Although the physical health of medical staff is slowly recovering after COVID-19 prevention and control policies are fully relaxed, people infected with COVID-19 will damage their mental health, which may last for a long time or even worsen. How these significant changes affect PTSD symptoms among health care workers remains unknown. To mitigate the negative impact of PTSD symptoms on health care workers**’** mental health, it is crucial to explore the prevalence of PTSD symptoms among frontline health care workers after COVID-19 control policies are fully relaxed. Therefore, the purposes of this study were to (1) investigate the prevalence of PTSD symptoms among health care workers after the full liberalization of COVID-19 prevention and control policy; (2) explore the relationship between PTSD symptoms and fear of COVID-19 and COVID-19 burden among health care workers. This study would further help health care managers develop effective psychological interventions to reduce the impact of COVID-19 infection on healthcare workers**’** mental health.

The traumatic events referred to in this study refer to patient rescue, death, isolation, and infection.

## Materials and methods

### Study design

Research team members conducted this multi-center cross-sectional survey between January 15 and January 16, 2023, in China. The COVID-19 pandemic has changed the methods of knowledge dissemination and data collection [30] and provided opportunities for the development of online platforms. The questionnaires were produced using Wenjuanxing (URL: https://www.wjx.cn/), the most widely used professional online questionnaire website. The QR code generated by Questionnaire Star is convenient for widespread dissemination. Members of this research group use WeChat groups, QQ groups, and other chat tools to distribute. The same IP address can only be submitted once; all questions must be completed before submission. The selection criteria for participants in this study were: 1) frontline medical staff during the COVID-19 pandemic; 2) able to read the questionnaire items and understand the purpose and content of the study; 3) have ever been in contact with infected patients, quarantined and Experience of being infected with COVID-19; 4) Sign the online electronic informed consent form.

### Data collection procedure

Health care workers who completed the questionnaire were encouraged to invite their colleagues to participate and forward QR codes in the study. The participants carefully completed the questionnaire and immediately submitted it via smartphones or tablets. The research team members strictly supervised daily online data collection to achieve the expected research objectives. A total of 1000 electronic questionnaires were distributed, and finally, 955 valid questionnaires were included for data analysis, with an effective recovery rate of 95.5%.

### Instruments

#### General characteristics

Participants were required to complete a questionnaire developed by research group members to provide information including gender, age, education level, marital status, technical title, the number of days working with illness, technical title, and working time.

#### PTSD

The PTSD-5 [10]is a widely used, efficient, and short 5-item assessment for PTSD experienced over the past month. Each item was scored from 0 to 1(0 = No; 1 = Yes), with a total score of ≥ 3 indicating “PTSD symptoms”. The Chinese version of the PC-PTSD-5 has been validated in Chinese family members of medical workers during COVID-19. The PC-PTSD-5 Chinese Version Cronbach’s alpha was 0.915 in the present study.

#### The fear of COVID-19

The fear of COVID-19 was assessed using the fear of COVID-19 Scale (FCV-19S) [31], which consists of 7 items. Each item was scored from 1 to 5(1 = “strongly disagree”, 2 = “disagree”, 3 = “neither disagree or agree”, 4 = “agree”, 5 = “strongly agree”), with the total score of ≥ 21 indicating “fear of COVID-19”. In the present study, the FCV-19S showed very high reliability, with Cronbach’s alpha of 0.86.

#### PHQ-2

Depressive symptoms were assessed using the validated 2-item Patient Health Questionnaire (PHQ-2), Chinese version. The total score of the PHQ-2 ranges between 0 and 6, with a higher total score indicating more severe depression, and a score of ≥ 3 was considered “depressive” [32]. In the current study, Cronbach’s alpha was 0.83.

#### GAD-2

Anxiety symptoms were evaluated using the validated Chinese version of the 2-item Generalized Anxiety Disorder Chinese version (GAD-2). The total score of the GAD-2 ranges between 0 and 6; higher total scores indicated more severe anxiety, and a score of ≥ 3 was considered “anxiety” [33]. In the current study, Cronbach’s alpha was 0.826.

#### Social support

Social support was measured using the Oslo Social Support Scale (OSSS-3). It consists of three items, with a total score of 3–14 points, of which 3–8 points are low social support, 9–11 points are moderate, and 12–14 are solid social support [34]. In the current study, Cronbach’s alpha was 0.85.

#### COVID-19 burden

COVID-19 burden was measured using the Scale developed by Nikunlaakso [35], which consists of two items: “Due to the situation of COVID-19, concerns about my health have made me fea” and “The COVID-19 situation has resulted in an escalation of my workload.” The answer is divided into yes or no; respondents who answered “yes” to both statements were classified as having a COVID-19 burden.

### Statistical analysis

Data analyses were performed using IBM Statistical Package for Social Science, Version 21.0 (SPSS Inc., Chicago, IL, USA). Demographic characteristics, PTSD symptoms scores, fear of COVID‐19, and depressive and anxiety symptoms are presented with mean, standard deviation (SD), numbers, and percentages. The chi-square test was used to compare differences in categorical variables between PTSD symptoms and non-PTSD symptoms groups. Binary logistic regression analysis was performed to analyze the factors associated with PTSD symptoms, and the ORs (odds ratios) and 95% CIs (confidence intervals) were calculated.

## Results

### Participant characteristics

Among 955 Chinese health care workers included in the data analysis, The age of the respondents ranged from 20 to 57 years old, with the mean age being (29.56 ± 6.13) years old. 178 (18.6%) were male, and 777 (81.4%) were female. 220 (23.0%) were unmarried, 107 (11.2%) were married but childless, and 618 (64.7%) were married and had children. Besides, 743 (78.5%) graduates were with bachelor**’**s degrees, 197 (20.8%) with master**’**s degrees, and 6(0.6%) with doctor**’**s degrees. Further socio-demographic information about this study is displayed in Table 1.

**Table 1:** Participants’ demographic information (*N* = 955)

Variable	Category	Participants	Percentage (%)
**Gender**	Male	178	18.6
	Female	777	81.4
**Marital status**	Unmarried	220	23
	Married but childless	107	11.2
	Married with children	618	64.7
	Other	10	1
**Age**	< 30	262	27.4
	30–39	523	54.8
	40–49	135	14.1
	≥ 50	35	3.7
**Occupation**	Doctors	259	27.1
	Nurses	696	72.9
**Hospital wards**	Outpatient clinic	84	8.8
	Emergency medicine	31	3.2
	Blood purification center	111	11.6
	Respiratory medicine	49	5.1
	Geriatrics	15	1.6
	ICU	60	6.3
	Obstetrics	36	3.8
	Pediatrics	48	5.0
	Surgery	160	16.8
	Gynecology	77	8.1
	Others	284	29.7
**Education**	Technical secondary school	13	1.4
	Junior college	116	12.1
	Undergraduate	641	67.1
	Postgraduate	160	16.8
	Doctorate and above	25	2.6
**Technical title**	Unrated	49	5.1
	Junior	407	42.6
	Intermediate	405	42.4
	Deputy senior	80	8.4
	High	14	1.5
W**orking time**	< 1 year	37	3.9
	1–2 years	58	6.1
	3–5 years	177	18.5
	6–10 years	239	25
	10–15 years	250	26.2
	> 15 years	194	20.3

### Factors associated with PTSD symptoms in the univariate analysis

In this study, the prevalence of PTSD symptoms among the health care workers was 24.3% (232/955). There were significant differences between the number of days working with illness, anxiety, depression, social support, the fear of COVID-19, and COVID-19 burden (*P* < 0.01, Table 2).

**Table2:** Characteristics of the participants based on the presence of PTSD symptoms (*N* = 955)

	**Non-PTSD symptoms** **(** ***n*** ** = 723)**	**PTSD symptoms** **(** ***n*** ** = 232)**	**χ2**	**P**
**Gender**			0.002	0.963
**Male**	135(75.8)	43(24.2)		
**Female**	588(75.7)	189(24.3)		
**Marital status**			5.860	0.110
**Unmarried**	174(79.1)	46(20.9)		
**Married but childless**	83(77.6)	(22.4)		
**Married with children**	456(73.8)	(26.2)		
**Other**	10(100.0)	(0.0)		
**Occupation**			2.292	0.130
**Doctor**	205(79.2)	54(20.8)		
**Nurse**	518(74.4)	178(25.6)		
**Age**			1.613	0.656
** < 30**	203(77.5)	59(22.5)		
**30–39**	396(75.7)	127(24.3)		
**40–49**	100(74.1)	35(25.9)		
** ≥ 50**	24(68.6)	11(31.4)		
**The number of days working with illness**			29.552	< 0.001
**0**	54(85.7)	9(14.3)		
**1–3 days**	225(82.7)	47(17.3)		
**4–6 days**	148(81.3)	34(18.7)		
** ≥ 7 days**	296(67.6)	142(32.4)		
**Working time**			8.155	0.148
** < 1 year**	32(86.5)	5(13.5)		
**1–2 years**	46(79.3)	12(20.7)		
**3–5 years**	139(78.5)	38(21.5)		
**6–10 years**	184(77.0)	55(23.0)		
**10–15 years**	175(70.0)	75(30.0)		
** > 15 years**	147(75.8)	47(24.2)		
**Technical title**			3.070	0.546
**Unrated**	41(83.7)	8(16.3)		
**Junior**	312(76.7)	95(23.3)		
**Intermediate**	303(74.8)	102(25.2)		
**deputy senior**	57(71.2)	(28.7)		
**high**	10(71.4)	4(28.6)		
**Anxiety**			102.832	< 0.001
**No**	407(90.6)	42(9.4)		
**Yes**	316(62.5)	190(37.5)		
Depression			105.238	< 0.001
**No**	387(91.7)	35(8.3)		
**Yes**	336(63.0)	197(37.0)		
**Social support**			32.276	< 0.001
**Low**	250(67.0)	123(33.0)		
**Middle**	344(78.5)	94(21.5)		
**High**	129(89.6)	15(10.4)		
**The Fear of COVID-19**			119.133	< 0.001
**No**	349(94.8)	19(5.2)		
**Yes**	374(63.7)	213(36.3)		
**COVID-19 burden**			73.287	< 0.001
**No**	344(90.3)	37(9.7)		
**Yes**	379(66.0)	195(34.0)		

### Binary analysis factors associated with PTSD symptoms

We put independent variables (*P* < 0.05) and dependent variables (grouping, 0 = non-PTSD symptoms group, 1 = PTSD symptoms group) into a binary logistic regression analysis model (Table 3). Factors affecting PTSD symptoms in health care workers are shown in Table 4. PTSD symptoms are more severe among health care workers with depression and anxiety (OR = 2.480, 95% CI 1.469–4.188; OR = 1.770, 95% CI 1.072–2.922). The more severe the fear of COVID-19, the higher the PTSD symptoms score (OR = 5.395, 95% CI 3.222–9.033). COVID-19 burden increased the risk of PTSD symptoms (OR = 2.517, 95% CI 1.659–3.819). The high level of social support was a protective factor for PTSD symptoms (OR = 0.458, 95% CI 0.242–0.866).

**Table 3:** Independent variable assignment

**Variables**	Assignment
**Depression**	No = 0
	Yes = 1
**Anxiety**	No = 0
	Yes = 1
**Social support**	Social support(Low) = 0
	Social support(Middle) = 1
	Social support(High) = 2
**The fear of COVID-19**	No = 0
	Yes = 1
**COVID-19 burden**	No = 0
	Yes = 1

**Table 4:** Binary logistic regression analysis of factors associated with PTSD symptoms

Variable	B	SE	Wald	*P*	OR	95% CI
Depression	0.908	0.267	11.544	0.001	2.480	1.469–4.188
Anxiety	0.571	0.256	4.980	0.026	1.770	1.072–2.922
Social support			6.440	0.040		
Social support(Middle)	-0.259	0.180	2.062	0.151	0.772	0.542–1.099
Social support(High)	-0.781	0.325	5.767	0.016	0.458	0.242–0.866
The fear of COVID-19	1.685	0.263	41.080	0.000	5.395	3.222–9.033
COVID-19 burden	0.923	0.213	18.844	0.000	2.517	1.659–3.819
Constant	-3.867	0.329	137.869	0.000	0.021	

## Discussion

PTSD symptoms among frontline health care workers has received increasing attention from scholars worldwide during the COVID-19 pandemic. As far as we know, this was the first study investigating the prevalence of PTSD symptoms and its related influencing factors among health care workers in China after the full liberalization of the COVID-19 prevention and control policy. In this cross-sectional study, we found that the prevalence of PTSD symptoms based on a total PC-PTSD 5 score of ≥ 3 was 24.3% among Chinese health care workers, consistent with the findings of Lee et al. [3], higher than the ratio of PTSD symptoms in youth [36] but lower than the Norway health care workers study by Johnson et al. and Huseyin [37]. About a fifth of frontline health care workers have reported PTSD symptoms after the full liberalization of COVID-19 control policies, possibly due to many reasons. In the current open policy environment, it is highly likely to contract the COVID-19 virus, resulting in an increasing number of infections. Consequently, the workload and challenges faced by medical professionals are bound to escalate. Moreover, this study indicates a slight rise with a rate of 24.3%. We must admit that direct comparisons with other studies should be cautious due to differences in the measurement tools used to assess PTSD symptoms and the cutoff values employed between studies. In Lee et al. (2023) [3], their subgroup analysis shows that the instrument and cut off used in this study, PC-PTSD (cut off of ≥ 3), is likely to produce higher estimates than other instruments. Psychological trauma experienced by HCWs during the COVID-19 pandemic can cause PTSD symptoms [38, 39]. In a study conducted in April–May 2020 by Dobson et al. [40], the prevalence of PTSD symptoms among Australian health workers was 29%. Studies conducted during previous coronavirus outbreaks (SARS, Middle East Respiratory Syndrome) had also found high rates of PTSD symptoms among health care workers, ranging from 9.4% to 47.8% [41–44]. However, in previous studies in India and Singapore, the incidence of PTSD symptoms among health workers during the COVID-19 pandemic was lower than in the current study, at 9.3 percent [14]. Differences in prevalence between countries can have many reasons; using different assessment questionnaires hinders firm conclusions. However, experts believe that the risk of PTSD symptoms is higher in Western countries because of the heightened concern about the potentially harmful effects of serious life events on mental health [45].

Studies have found that health care workers have higher levels of worry and PTSD symptoms because they work directly with infected people [42, 46, 47]. Studies have found that the more multiple traumatic events experienced, the greater the risk of developing PTSD symptoms [48, 49]. However, accurate comparisons are difficult because some other studies used different questionnaires.

In addition, the study found that COVID-19 fear is associated with PTSD symptoms, which is consistent with previous literature [50]. This may be because health care workers facing COVID-19 infection develop COVID-19 fear, psychological distress, and severe concern about recovering their physical health [51]. COVID-19 has hit people worldwide hard due to its rapid spread and high mortality rates, leading individuals to fear COVID-19 [52]. Studies have shown that fear of contracting COVID-19 and concerns about one**’**s health are associated with poor mental health in individuals [53]. Studies have shown that fear of COVID-19 is associated with work stress and emotional exhaustion [54], and when emergency nurses**’** COVID-19 fear increases, their personal accomplishment and mental health decline accordingly. Fear of COVID-19 is a common predictor of PTSD symptoms, suggesting that mental health services for health care workers may help prevent future PTSD symptoms by alleviating their fear of COVID-19. This study suggests that reducing health care workers**’** fear of COVID-19 may be an excellent strategy for reducing PTSD symptoms during the COVID-19 pandemic. Therefore, during the COVID-19 pandemic, governments and administrators should take COVID-19 fears among healthcare workers seriously. The exact mechanism between the fear of COVID-19 and PTSD symptoms needs further study.

### COVID-19 burden

As expected, the COVID-19 burden is associated with PTSD symptoms among health care workers. As a public health emergency, the suddenness, unpredictability, development uncertainty, and complexity of COVID-19 have caused public panic or anxiety, affected people**’**s everyday lives, work order, and social stability [55, 56], led to changes in people**’**s lifestyles and increased psychological burden, and further aggravated the occurrence of PTSD symptoms. In addition to the common contributing factors, we included in our study the burden that the COVID-19 pandemic has placed on health care workers. A Finnish study showed that frontline COVID-19 work is associated with psychological distress among workers [57]. Ervasti et al. [58] studied the effects of COVID-19 on psychosocial stressors. COVID-19 has dramatically affected the life and health of healthcare workers, increasing their work pressure and psychological burden. Based on the particularity of the medical personnel group, the government should set up some targeted special measures to protect this group, minimize their psychological burden, and ensure the physical and mental health of medical workers.

As expected, anxiety and depression were positively associated with PTSD symptoms, similar to the previous study [59]. Studies have shown that quarantine and other national emergencies can lead to stress and mental disorders such as PTSD symptoms [60, 61]. Nikayin et al. found that PTSD symptoms were strongly associated with post-ICU anxiety [62] and post-critical illness comorbid depression [63] and that anxiety can lead to PTSD symptoms [64]. The development of PTSD symptoms depends on the type of event exposure, its intensity, and its frequency [65]. With the COVID-19 pandemic, health care workers need to be on the front lines of the fight against the pandemic. The poor working conditions and the large number of cases and deaths heavily burden healthcare workers [66]. Health care workers are working on the front lines of the fight against the COVID-19 pandemic, having close daily contact with people with COVID-19, facing more significant mental stress, and are more prone to mental health issues [67]. Health care workers have been found to show higher levels of anxiety and depression compared with other populations [68, 69]. We assume that the stressful environment specific to medical work, combined with prolonged isolation, separation from family members, and concerns about the health of family members, has led to anxiety and depression among healthcare workers, further aggravating their PTSD symptoms levels. Lee et al. [70] found that greater stress on family and social relationships was a risk factor for psychological distress. The Covid-19 pandemic and infectious disease control measures such as lockdowns have caused psychological distress for health care workers and their families [71] and also have had a severe impact on the family lives of the subjects [72].

Therefore, state and hospital administrators need to pay special attention to the mental health of medical staff and provide them with psychological support and counseling.

This study found that social support was a protective factor in PTSD symptoms among health care workers. Work on the front lines had a huge physical and psychological impact on health care workers, increasing their mental illness risk. The study found that the nurses who received more family support had fewer psychological problems such as anxiety and depression [73]. In 2004, WHO identified social support as one of the critical social determinants of health [74]. Social support can help reduce stress and provide appropriate ways to deal with it [75]. Low social support has been found to be a contributing factor to depression [76]. Studies have found that good social support increases well-being, reduces stress and burnout associated with the work environment, and improves job satisfaction [77] and sleep quality. There is a greater emphasis on collectivism and the family in Asian cultures, so the family is the primary source of support [78]. Strengthening social support can effectively reduce the mental health risks associated with stress. Overall, social support can positively impact PTSD symptoms by evoking positive feedback from individuals at the psychological and cognitive levels, such as hope, positive reevaluation, self-efficacy, self-esteem, and a sense of community. Policymakers and managers should consider developing targeted social support policies to help reduce PTSD symptoms in healthcare workers.

### Limitations

The strengths of this cross-sectional study include the large sample size, the multi-center design, and the standardized and proven PTSD symptoms screening instruments. However, This study has several limitations. First, the survey is voluntary, so health care workers may choose to participate in this study because of their mental health issues, which may increase or decrease the prevalence rates of our survey. Second, the PC-PTSD 5 scale was used in this study to screen the PTSD symptoms of health care workers, which was not a clinical diagnostic instrument. Third, due to the cross-sectional study, the causal relationship between PTSD symptoms and other variables (depression, anxiety, fear of COVID-19.) cannot be established. In the future, more longitudinal studies are needed to understand the changing patterns of PTSD symptoms among healthcare workers infected with COVID-19.

## Conclusion

In summary, health care workers showed severe PTSD symptoms three years after the COVID-19 outbreak. Anxiety, depression, COVID-19 fear, and COVID-19 burden were all significantly associated with PTSD symptoms, and social support is a protective factor for PTSD symptoms. Because of the negative impact of PTSD symptoms on the quality of medical care and quality of life for frontline health care workers, health boards and policymakers should develop effective interventions to reduce PTSD symptoms for health care workers.

## Data Availability

The datasets in the current study of this study are available from the corresponding author on reasonable request.
